# Does sensitization by SARS-CoV-2 immune complexes trigger DRESS syndrome?

**DOI:** 10.1016/j.bjid.2022.102337

**Published:** 2022-02-28

**Authors:** Virgínia Barbeitos Cruz, Luiz Fernando Fróes Fleury Júnior, Christiane Reis Kobal, Nilzio Antonio da Silva

**Affiliations:** a Health Sciences Program, School of Medicine, Universidade Federal de Goiás, Goiânia, GO, Brazil; b Clinical Hospital, School of Medicine, Universidade Federal de Goiás, Goiânia, GO, Brazil; c Department of Infectious Diseases, Hospital of Tropical Diseases of Goiás, Goiânia, GO, Brazil

**Keywords:** COVID-19, SARS-CoV-2, Exanthema, Drug reaction with eosinophilia and systemic symptoms (DRESS) syndrome

## Abstract

The diagnosis of coronavirus disease (COVID-19) has been a great challenge since the infection affects not only the respiratory system, but also different organs, given the intense inflammatory and autoimmune reaction triggered by severe acute respiratory syndrome coronavirus 2 (SARS-CoV-2). Herein we present a case of a 36-year-old male patient, with some comorbidities and previous use of carbamazepine, who developed a severe condition triggered by COVID-19, including extensive exfoliative erythroderma and severe impairment of liver function, which lasted approximately 80 days.

## Introduction

Extra-respiratory manifestations of severe acute respiratory syndrome coronavirus 2 (SARS-CoV-2) infection have been described and are especially important for diagnostic and epidemiological purposes, once most infected people are asymptomatic.^1^^,^^2^ Among these manifestations, dermatoses have been observed in approximately 20.4% of the patients diagnosed with coronavirus disease (COVID-19), associated with changes caused by the virus itself or by worsening underlying dermatological conditions.^2^

Cutaneous manifestations related to COVID-19 have not been frequently reported, likely due to underdiagnosis. However, so far, six large groups of possible lesions have been described: maculopapular rash, urticaria, chilblain, vesicular lesions, livedo reticularis, and petechiae.^3^ Some idiosyncratic reactions to the drugs used to treat COVID-19 have also been reported. In these cases, the possible mechanisms involved could be lymphocytic vasculitis induced by viral particles, deposition of immune complexes, or skin microvessel thrombosis. Erythema, urticaria, contact dermatitis, varicella-like rash, erythroderma, livedo reticularis, lichenoid photodermatitis, and acute generalized exanthematous pustulosis are among the reported reactions.^1^^,^^2^

Gastrointestinal manifestations have also been found in 79% of the patients with COVID-19, although their pathophysiology remains poorly understood. The large expression of angiotensin-converting enzyme 2 on the gastric, duodenal, and rectal epithelia glandular cells, as well as on the endothelial cells of the small intestine, is thought to be one of the mechanisms involved. The most frequent symptoms are anorexia, abdominal pain, nausea, vomiting, diarrhea, and gastrointestinal bleeding.^4^, ^5^, ^6^

About one third of the patients infected with SARS-CoV-2, especially males, between 35 and 56 years old, have liver dysfunction. Pre-existing liver diseases, elevated alanine aminotransferase and transaminase levels, thrombocytopenia, and hypoalbuminemia have been pointed out as risk factors for the tissue damage observed in these individuals. Intense inflammatory reaction and autoimmune sensitization, mediated by toll-like receptors and killer T lymphocytes, are among the possible pathogenic mechanisms.^7^^,^^8^

Herein we present a case of a young, obese, male patient undergoing alcohol addiction treatment, diagnosed with COVID-19 due to community transmission, with quickly serious gastrointestinal manifestations, severe impairment of liver function, in addition to exuberant dermatologic manifestations, with no respiratory symptoms.

### Case report

On April 1, 2020 [Day 1 (D1)], a 36-year-old male patient, with several comorbidities (obesity, hepatic steatosis, hypertension, panic disorder, and alcohol addiction), and under treatment with carbamazepine (400 mg/day), escitalopram (20 mg/day), amlodipine (50 mg/day), losartan (50 mg/day), and nebivolol (5 mg/day), sought for assistance in private practice due to the following symptoms: mild to moderate abdominal pain, diffuse, associated with dyspepsia, belching, with no nausea, vomiting, or fever. On D3, the pain worsened and was described by the patient as left thoracolumbar colic, radiating to the periumbilical region and testicles, associated with important abdominal distension. He was checked in the hospital with normal temperature, preserved peristalsis, no change in urinary or intestinal functions, non-nodular liver, palpable at 3.0 cm from the costal margin, and non-palpable axillary, inguinal, or cervical lymph nodes. In addition to oral rehydrating solutions, the prescribed treatment included fexofenadine hydrochloride (180 mg/day), paracetamol (1,500 mg/day), dipyrone (1,000 mg/day), codeine (30 mg/day), and scopolamine solution + dipyrone (30 mg + 750 mg/day). On the same day, he underwent total abdomen computed tomography (CT) that revealed hepatic steatosis, kidney stones, bowel loops normal in diameter, normal peritoneum, retroperitoneum, gall bladder, and bile ducts.

On D7, a cholangioresonance confirmed hepatic steatosis and showed that the liver was slightly enlarged, with sparse infracentimetric cysts, adenomyomatosis at the bottom of the gall bladder, intra and extrahepatic bile ducts with no dilations, normal pancreas, pancreatic ducts, and spleen, normally positioned kidneys, bilateral single kidney microcysts, calyceal system with no dilations, single lymph node in the hepatic hilum (1.4 cm × 1.4 cm). On D8, the molecular diagnosis of COVID-19 was confirmed.

**Fig. 1:**
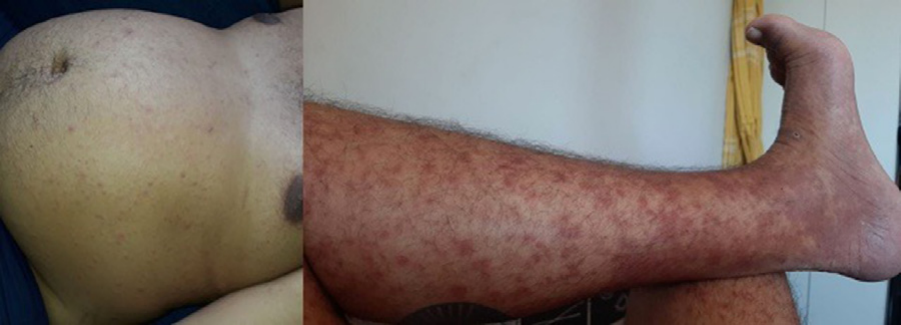
In the initial phase of the condition, erythroderma and digestive manifestations predominated, with abdominal distention, hepatomegaly, and diarrhea.

**Fig. 2:**
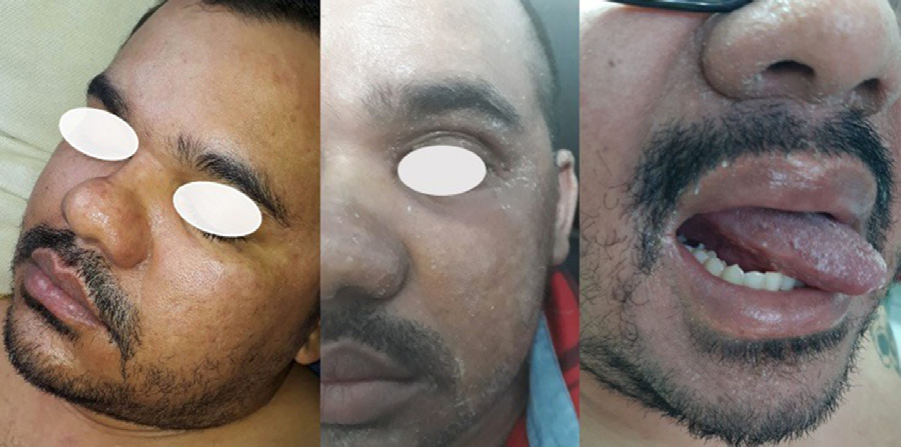
Facial edema and enlargement of the nasal wings, lips, and tongue were present both in the erythematous phase and in the desquamative phase.

**Fig. 3:**
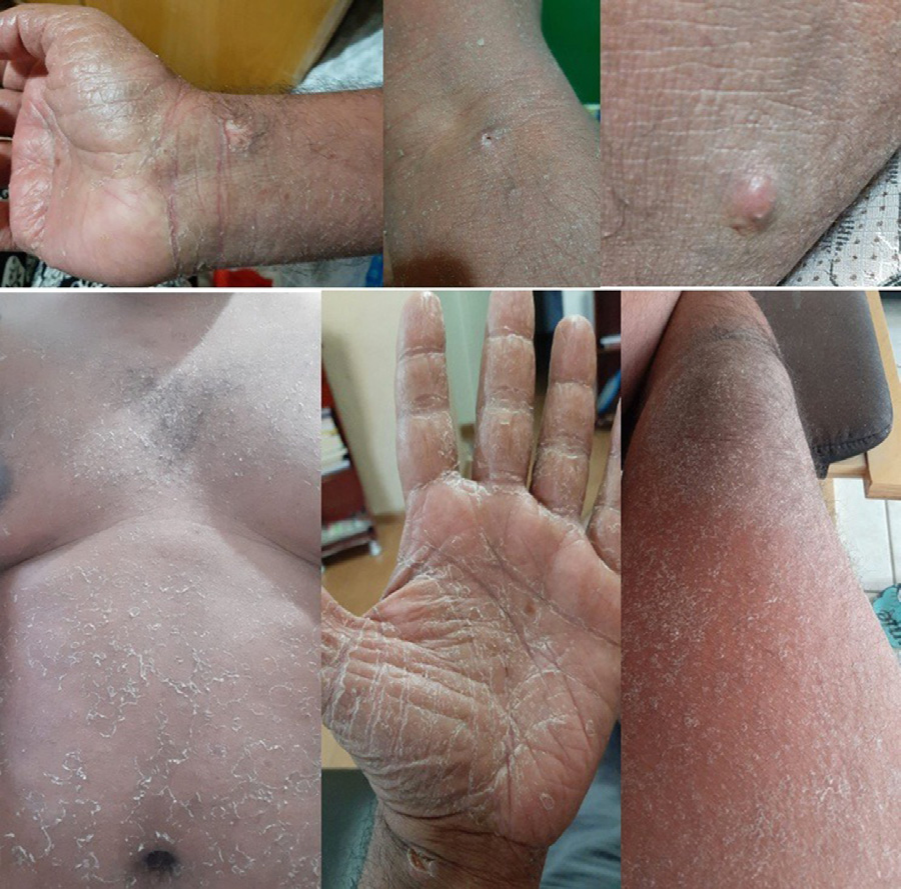
Exfoliation reached the entire skin, including scalp, palms of the hands and soles of the feet, and was associated with sparse pustules and hyperpigmentation, especially in skin fold regions.

From D8 to D14, the patient had lip fissures, painful and numerous aphthous ulcers that limited food intake, flatulence, diarrheal white stools, with no jaundice, anal fissures, and hemorrhoid with droplets of blood (Fig. 1). A treatment with lidocaine ointment (50 mg) in association with hydrocortisone acetate (2.5 mg), zinc oxide (180 mg), aluminum subacetate (35 mg), and triamcinolone acetonide (1 mg) was prescribed. On D11, he complained about loss of taste, pronounced edema of the face and limbs (Fig. 2). On D12, these gastrointestinal symptoms were followed by dermatological manifestations, although the patient had no history of dermatoses. From D12 on, monomorphic vesicles dispersed in the trunk and limbs were observed as a first alteration, evolving to maculopapular rash, initially erythematous and delimited, then violet and coalescent, also affecting the palms of the hands and the soles of the feet, associated with desquamation, sparse fissures, and burning pain (Fig. 3). On D29, these dermatological manifestations reached such a proportion that the patient was transferred to the semi-intensive care unit, following the isolation protocol. At this moment, the hospital team was dealing with the possibility of toxidermia, which they attributed to the use of morphine, and the patient was started on prednisolone (40 mg/day).

The patient was discharged from the hospital in a better condition on D35. However, on D37, he had recurrence of the cutaneous lesions, then aggravated, presenting with putrid odor on the skin, sparse pustules, and chills when the axillary temperature reached 38.9°C. Skin culture was positive for *Staphylococcus aureus* and fungal infection was ruled out. On D37, the patient started treatment with sulfamethoxazole and trimethoprim (400 mg + 80 mg/day) for 10 days.

Periodical complete blood counts revealed: red series with no changes either in count or in shape throughout the clinical course; eosinophilia (1,132/µL on D8 and 3,198/µL on D14) in the most acute phase of the rash; mild leukocytosis (13,610/µL), with a shift to the left, during staphylococcal co-infection and corticosteroid administration; gamma-glutamyl transferase, 309–590 U/L; alanine aminotransferase, 113–574 U/L; aspartate amino transferase, 31–89 U/L; alkaline phosphatase, 162–169 U/L; increased serum C-reactive protein (2.95–3.40 mg/dL); bilirubins, glucose, lipase, amylase, calcium, magnesium, and creatine phosphokinase remained normal throughout the clinical evolution. Tests for dengue, hepatitis A, hepatitis B, cytomegalovirus, syphilis, and AIDS were non-reactive. The attempt to taper off corticosteroid therapy worsened the dermatological condition, with lichenification, pigmentation, and skin fissures over the joints. Weight loss, loss of muscle mass, generalized myalgia, weakness, and fasciculation in the limbs made walking difficult. The magnitude of the skin lesions and the acute hepatitis in the presence of SARS-CoV-2 infection, in addition to the use of carbamazepine, led us to consider overlapping pharmacotoxicity. Therefore, carbamazepine discontinuation started on D43, 2020, with marked improvement of the rash and progressive skin recovery. The patient did not show any respiratory changes since the onset of symptoms, maintaining O_2_ saturation around 98%.

On D49, COVID-19 IgG/IgM was non-reactive. The treatment finished on D80 of symptom onset, with normalization of liver and kidney functions, great weight loss, and gradual recovery of activities of daily living. Skin biopsy performed on D111, already in the recovery phase from illness, revealed medication-induced spongiotic dermatitis.

## Discussion

COVID-19 has been considered a systemic disease, capable of generating breakdown and rapid exhaustion of defense and autoimmunity mechanisms, with the most diverse degrees of involvement of different tissues and systems other than just the respiratory system, and often dissociated from it.^6^^,^^9^ In the present report, the first manifestation of SARS-CoV-2 infection was abdominal pain, radiating to the lumbar region and testicles, suggesting movement of kidney stone, not confirmed by imaging exams. Similarly, another report described the case of a patient who was referred to the emergency department due to abdominal and testicular pain and was subsequently diagnosed with COVID-19.^10^

Fever was not a striking sign of acute infectious disease in our patient. In a review article, 18.7% of the patients diagnosed with COVID-19 and admitted to hospital did not have fever.^11^ As observed in another study carried out in Wuhan (China), 62.4% of those infected with COVID-19 who presented with gastrointestinal manifestations alone or associated with respiratory impairment had no fever.^12^ The patient of this case report had fever only during the staphylococcal skin infection.

Our patient's previous history of hepatic steatosis is relevant, even though his liver functional parameters were stable until SARS-CoV-2 infection. Intrahepatic cholestasis may have been caused by peri-hilar lymph node compression identified in imaging exam, inflammation, and/or idiosyncrasy related to the use of carbamazepine.

Metabolized by the CYP450 system, drugs can produce toxic metabolites, due to enzymatic failure (epoxide hydrolase), and trigger a severe hypersensitivity syndrome, with consequent apoptosis and magnification of autoimmune response, named drug rash with eosinophilia and systemic symptoms (DRESS) syndrome. The estimated incidence of DRESS syndrome, or drug-induced hypersensitivity syndrome (DiHS)/DRESS, as proposed by the Registry of Severe Cutaneous Adverse Reaction (RegiSCAR) Consensus,^13^ ranges from 1:1000 to 1:10,000 exposures.^14^^,^^15^ This syndrome is characterized by skin manifestations that include maculopapular rash on the face and limbs, erythroderma, face edema, high fever (38–40°C), marked eosinophilia (95%), lymphadenopathy, liver involvement (50–93.8% of patients) causing hepatitis and cholestasis, nephritis, influenza-like symptoms, pneumonitis, myocarditis, and colitis. The difficult differential diagnosis, especially with infectious diseases, increases mortality to 5–10% of the patients due to liver failure.^14^, ^15^, ^16^, ^17^ Over 60% of the DRESS syndrome cases are associated with viral infection, especially human herpesvirus 6 and 7, Epstein Barr virus, and human cytomegalovirus.^18^

Numerous drugs from different categories have been involved in the DRESS syndrome, and its incidence seems to have been aggravated during the current pandemic. In a review of medical records of patients hospitalized at San Raffaele Hospital, Milan, Italy, diagnosed with COVID-19 during the first three waves, a 340-fold increase in the diagnosis of this syndrome was identified compared to pre-pandemic numbers. Hydroxychloroquine, lopinavir-ritonavir, and β-lactam antibiotics were the most likely associated drugs.^19^

A case of intense rash was reported in a patient diagnosed with COVID-19 in an intensive care unit associated with eosinophilia (950/µL) and altered renal and hepatic functions. The patient had been administered azithromycin, hydroxychloroquine, heparin, propofol, clonidine, norepinephrine, sufentanil, rocuronium, pantoprazole, sevoflurane, cefuroxime, and flucloxacillin prior to cutaneous manifestations. Skin biopsy showed moderate lymphohistiocytic and eosinophilic perivascular infiltrate. The diagnosis of DRESS syndrome was considered, and azithromycin and hydroxychloroquine were immediately discontinued. Clinical resolution was progressively reached after 15 days of corticosteroid therapy, and the patient was discharged within three weeks.^20^

A young woman has sought care in the emergency room with an extensive pruritic rash, fever, lymphadenomegaly, arthralgia, edema of face and limbs, associated with eosinophilia and leukocytosis. She had had a previous diagnosis of ulcerative colitis treated with sulfasalazine 14 days before the onset of the other symptoms. Routine RT-PCR evidenced SARS-CoV-2 infection.^21^

The severe cutaneous manifestation observed in our patient lasted 68 days, while the average duration related exclusively to COVID-19 is 10 days.^2^ The idiosyncratic manifestations of carbamazepine, one of the drugs most often associated with the DRESS syndrome, generally last between three weeks and three months, with an average of eight weeks from the beginning of the exposure, sometimes precipitated by viral infection.^14^ Our patient started using this drug three months before symptom onset. Recognition of the toxicity of carbamazepine, and its withdrawal, was delayed, which we attribute to the confusion generated by the great mimic power of SARS-CoV-2. The skin biopsy, performed in the period of symptom remission, revealed spongiotic lesions, one of the patterns observed in a study carried out in Italy,^22^ although nonspecific. According to the RegiSCAR scoring system for classifying DRESS cases, our patient reached grade 6, i.e. a definite case.^23^ It is also worth mentioning that, prior to the onset of the dermatological condition, our patient was using paracetamol and dipyrone, drugs that are also involved in immediate or acute hypersensitivity reactions.^24^

Seroconversion to IgM and IgG was observed within a period of nine days of symptom onset,^25^ a fact not observed in the present patient, since IgG remained negative after 45 days. This result alerted us to the sensitivity of the available tests, a critical element in the pandemic context. Two female members of the patient's family, close to him, young and healthy, were monitored by telemedicine in home isolation for 14 days. Both reported taste loss, weakness, and holocranial headache, and tested negative for COVID-19.

Detailing of the exuberant digestive and dermatological manifestations experienced by our patient aimed to contribute to the construction of a clinical standard, not yet established for COVID-19, as well as to alert health professionals to the necessary rigor of biosafety measures, given the multiplicity of symptoms and tissues affected by SARS-CoV-2. It was also our goal to reinforce the need for standardization of diagnostic nomenclature of toxidermias.

## Funding

This research received no specific grant from any funding agency in the public, commercial, or not-for-profit sectors.

## Ethics approval and consent to participate

Not applicable.

## Consent for publication

Written informed consent was obtained from the patient.

## Conflicts of interest

The authors declare no conflicts of interest.
